# *FTO* Genotype and Type 2 Diabetes Mellitus: Spatial Analysis and Meta-Analysis of 62 Case-Control Studies from Different Regions

**DOI:** 10.3390/genes8020070

**Published:** 2017-02-11

**Authors:** Ying Yang, Boyang Liu, Wei Xia, Jing Yan, Huan-Yu Liu, Ling Hu, Song-Mei Liu

**Affiliations:** 1 Center for Gene Diagnosis, Zhongnan Hospital of Wuhan University, Donghu Road 169#, Wuhan 430071, China; yangying0109@whu.edu.cn; 2 Department of Geography, Wilkeson Hall, State University of New York at Buffalo, Buffalo, NY 14261, USA; bliu24@buffalo.edu; 3 Department of Clinical Laboratory, Wuhan Children’s Hospital (Wuhan Maternal and Child Healthcare Hospital), Tongji Medical College, Huazhong University of Science & Technology, Wuhan 430016, China; 18971319110@163.com; 4 Hubei Meteorological Information and Technology Support Center, Wuhan 430074, China; yanjing619@hotmail.com; 5 Department of Clinical Medicine, Hubei University of Medicine, Hubei 442000, China; gutentag95@sina.com; 6 Department of Neurology, Wuhan Children’s Hospital (Wuhan Maternal and Child Healthcare Hospital), Tongji Medical College, Huazhong University of Science & Technology, Wuhan 430016, China; m18372622675@163.com

**Keywords:** type 2 diabetes mellitus, T2DM, fat mass and obesity-associated, *FTO*, polymorphism(s), spatial analysis, meta-analysis

## Abstract

Type 2 diabetes mellitus (T2DM) is a global health problem that results from the interaction of environmental factors with genetic variants. Although a number of studies have suggested that genetic polymorphisms in the fat mass and obesity-associated (*FTO*) gene are associated with T2DM risk, the results have been inconsistent. To investigate whether *FTO* polymorphisms associate with T2DM risk and whether this association is region-related, we performed this spatial analysis and meta-analysis. More than 60,000 T2DM patients and 90,000 controls from 62 case-control studies were included in this study. Odds ratios (ORs), 95% confidence intervals (CIs) and Moran’s I statistic were used to estimate the association between *FTO* rs9939609, rs8050136, rs1421085, and rs17817499, and T2DM risk in different regions. rs9939609 (OR = 1.15, 95% CI 1.11–1.19) and rs8050136 (OR = 1.14, 95% CI 1.10–1.18) conferred a predisposition to T2DM. After adjustment for body mass index (BMI), the association remained statistically significant for rs9939609 (OR = 1.11, 95% CI 1.05–1.17) and rs8050136 (OR = 1.08, 95% CI 1.03–1.12). In the subgroup analysis of rs9939609 and rs8050136, similar results were observed in East Asia, while no association was found in North America. In South Asia, an association for rs9939609 was revealed but not for rs8050136. In addition, no relationship was found with rs1421085 or rs17817499 regardless of adjustment for BMI. Moran’s I statistic showed that significant positive spatial autocorrelations existed in rs9939609 and rs8050136. Studies on rs9939609 and rs8050136 focused on East Asia and South Asia, whereas studies on rs1421085 and rs17817499 were distributed in North America and North Africa. Our data suggest that the associations between *FTO* rs9939609, rs8050136 and T2DM are region-related, and the two single-nucleotide polymorphisms contribute to an increased risk of T2DM. Future studies should investigate this issue in more regions.

## 1. Introduction

Diabetes is a growing global health problem; more than 300 million people live with diabetes worldwide [1], and the prevalence of diabetes is estimated to rise [2]. Type 2 diabetes mellitus (T2DM) is the most common type of diabetes, as it accounts for more than 90% of diabetes cases [3]. Although the pathogenesis mechanisms of T2DM have not been clearly defined, a combination of genetic and environmental factors is believed to lead to the disease [4].

The fat mass and obesity-associated (*FTO*) gene is located on chromosome 16 (16 q12. 2), containing nine exons and several single-nucleotide polymorphisms (SNPs) [5]. In 2007, a genome-wide association study (GWAS) searching for type 2 diabetes-susceptibility genes confirmed a common variant (rs9939609) in the *FTO* gene that predisposes European populations to diabetes [6]. Since then, a large number of studies have focused on the association between *FTO* polymorphisms, expression and T2DM in different populations [7,8,9,10]. Meanwhile, some meta-analyses have been performed to elucidate the relationship between *FTO* polymorphisms and T2DM risk. For instance, a meta-analysis utilizing data from studies prior to 2010 identified an association between rs9939609 and T2DM in East and South Asians [11]. Additionally, a Norwegian population-based Nord-Trøndelag Health Study (HUNT study) [12], including three cohorts (HUNT, Malmö Diet and Cancer (MDC) and Malmö Preventive Project (MPP)), reported strong association between rs9939609 and T2DM risk in Scandinavians after adjustment for age, sex and body mass index (BMI). Another meta-analysis of association between obesity/BMI-associated loci and T2DM risk [13], using data from studies conducted between 2007 and 2012, revealed that *FTO* rs9939609 significantly associated with T2DM which also remained significant following adjustment for BMI; Analysis by Vasan et al. [14] has provided evidence that rs9939609 is associated with obesity and T2DM in Asian Indians, with modest attenuation observed when adjusting for BMI. These and the majority of other previous meta-analyses have focused on single population or one *FTO* loci without consideration of population-specific environmental influences among different regional subgroups. As such, the results of these meta-analyses cannot be generalized to the world.

More recently, geographic information systems (GIS) and spatial analysis are increasingly applied in the investigation of disease spatial pattern, including diabetes [15].

To more comprehensively clarify the association between *FTO* polymorphisms and T2DM risk, we performed this spatial analysis and meta-analysis to include most, if not all, eligible studies published before January 2017.

## 2. Materials and Methods

### 2.1. Search Strategy

Eligible articles were selected by searching up to January 2017 in PubMed and EMBASE using the following keywords: “*FTO* or fat mass and obesity-associated gene” and “variant or variation or polymorphism” and “type 2 diabetes or type 2 diabetes mellitus or T2D or T2DM”. Articles obtained from the initial search were then screened based on the inclusion criteria described below. Only publications with English language were included. If more than one population was included in a given article, results were considered as separate studies.

### 2.2. Study Selection Criteria and Data Extraction

The selected studies met all of the following inclusion criteria. The studies had to: (1) evaluate the association between *FTO* polymorphisms and T2DM risk; (2) have a case–control or cohort design; and (3) provide odds ratios (OR) with a 95% confidence interval (CI) or sufficient data for calculation. From each study, the following information was collected: (1) name of the first author; (2) year of publication; (3) country of origin; (4) ethnicity of the samples; (5) sample size of cases and controls; (6) Hardy–Weinberg equilibrium (HWE) in control groups; and (7) data of SNPs. Data were independently extracted from eligible articles by two authors (YY and HYL) according to the criteria described. Discrepancies were resolved by discussion with a third reviewer (SML), and a consensus approach was used.

### 2.3. Spatial Analysis

The ArcGIS v10.3 software is a GIS tool that has become increasingly prevalent in public health research to understand the spatial pattern of diseases and genetic biodiversity [15]. This software was utilized to depict the geographic distribution of the association studies. R was used to calculate Moran’s *I*, a statistic for evaluating the spatial autocorrelation [16,17]. By constructing the spatial weight matrix, Moran’s *I* coefficient can be calculated as follows:

$$I=\;\frac{N}{\sum_{i}\sum_{j}w_{ij}}\frac{\sum_{i}\sum_{j}w_{ij}\left(X_{i}-\bar{X}\right)\left(X_{j}-\bar{X}\right)}{\sum_{i}{\left(X_{i}-\bar{X}\right)}^{2}}$$

*N* is the number of spatial units indexed by *i* and *j*; *X* is the variable of interest; $\bar{X}$ is the mean of X; and $w_{ij}$ is an element of a matrix of spatial weights. In this study, we constructed the spatial weight matrix by making a distance threshold *h*. If the distance between point *i* and point *j* is smaller than h, $w_{ij}$ will be 1. Otherwise, $w_{ij}$ will be 0. It is worth noting that all diagonal elements of matrix w are all 0. Monte Carlo simulations were used to test for the significance of Moran’s *I*.

### 2.4. Statistical Analysis

The strength of association between *FTO* SNPs and T2DM risk was expressed as a pooled OR and 95% CI. A *z*-test was performed to evaluate the significance of the pooled OR (*p* < 0.05 was considered statistically significant). The χ^2^-test-based Q test and *I*^2^ were performed to assess the heterogeneity of the studies. A value of *I*^2^(%) > 50% or *p* ≤ 0.10 indicated significant heterogeneity. A random-effects model (DerSimonian–Laird method) [18] was used to determine the pooled OR in the presence of heterogeneity; otherwise a fixed-effects model (Mantel–Haenszel method) [19] was used. Subgroup analyses were performed by region. Sensitivity analyses were performed to assess the stability of the combined results by excluding the studies with unknown HWE in controls. Publication bias was evaluated by Begg’s test [20] and Egger’s test [21] (*p* < 0.05 was considered statistically significant). Data analyses were conducted using STATA 12.0 (Stata-Corp LP, College Station, TX, USA).

## 3. Results

### 3.1. Study Characteristics and Quality

**Figure 1:**
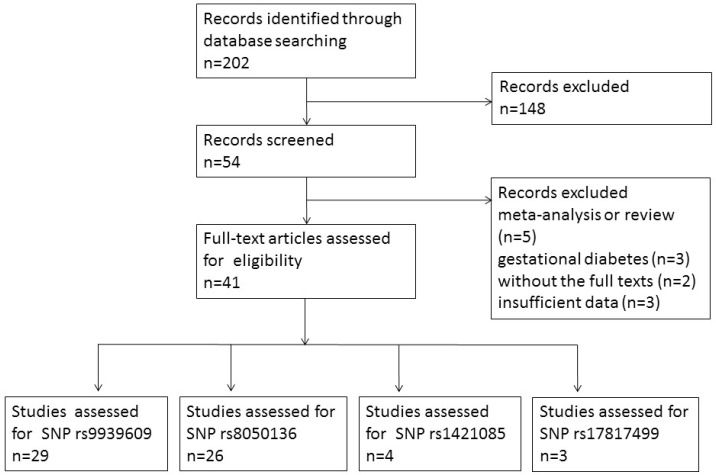
Study selection flow chart based on preferred reporting items for spatial analysis and meta-analysis.

A total of 202 potentially relevant papers were identified from PubMed and EMBASE. After reading the title and abstract, 148 articles were excluded because they addressed topics that did not match the inclusion criteria. The full texts of the remaining 54 articles were carefully screened. We excluded five meta-analyses or reviews, three articles that explored the association between *FTO* polymorphisms and gestational diabetes, two articles that did not include the full text, and three papers with insufficient data. In total, 41 articles met the inclusion criteria. A flow chart describing the article selection for our meta-analysis is shown in Figure 1. Of the articles included, 29 studies investigated rs9939609, 26 studies explored rs8050136, four studies investigated rs1421085 and three studies explored rs17817499. Other SNPs that were assessed in only one study were not analyzed. The detailed characteristics of the included studies are shown in Table 1.

### 3.2. Region-Related Associations Exist between rs8050136, rs9939609 and T2DM

**Figure 2:**
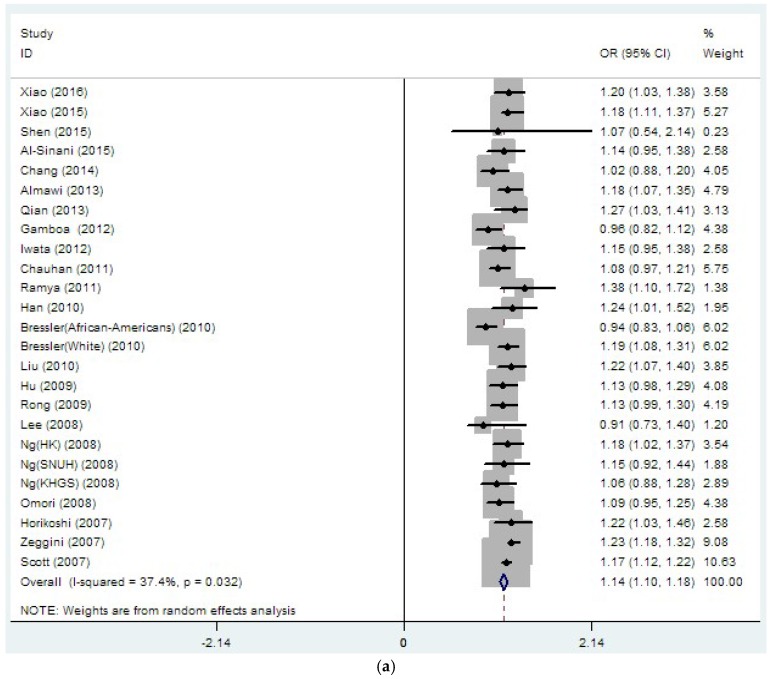

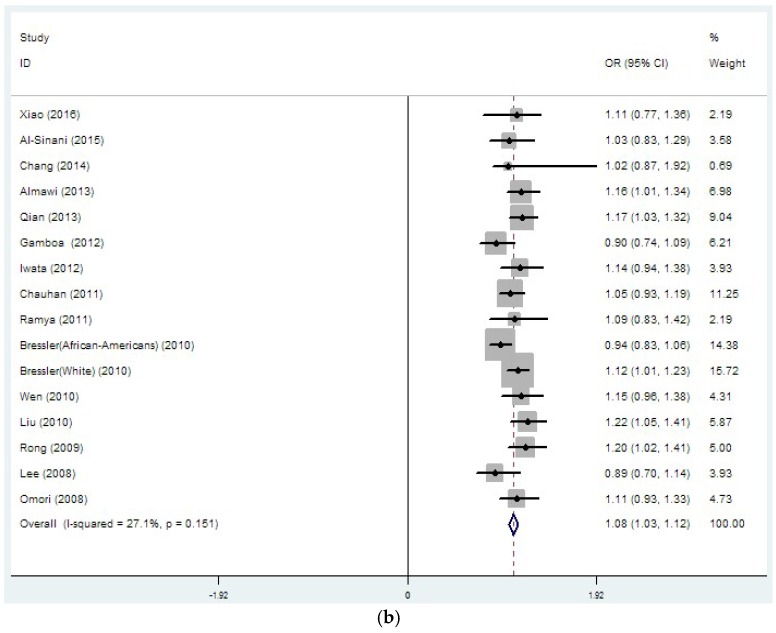
Meta-analysis for the associations between rs8050136 and Type 2 diabetes mellitus (T2DM) risk: (**a**) without; and (**b**) with adjustment for body mass index (BMI).

**Figure 3:**
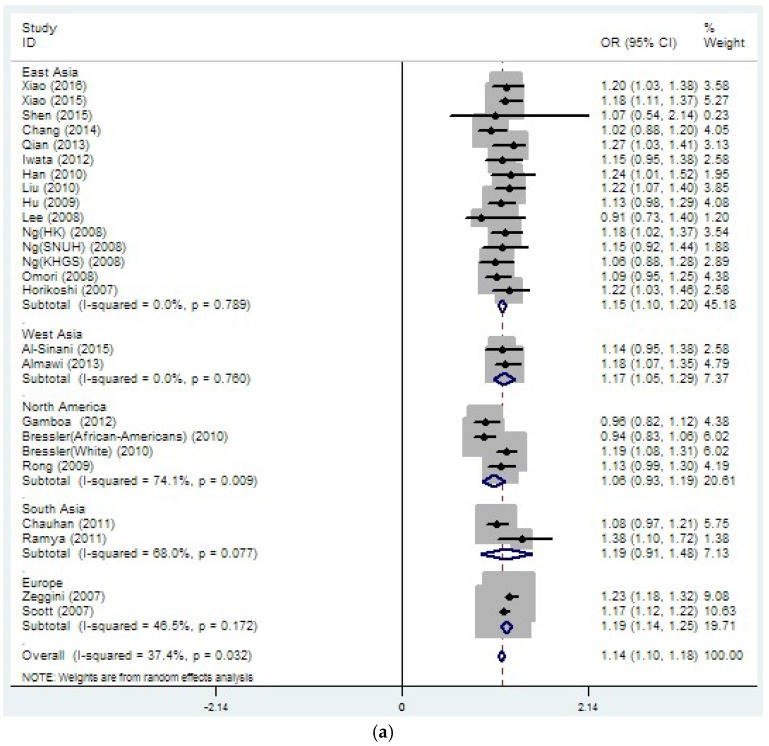

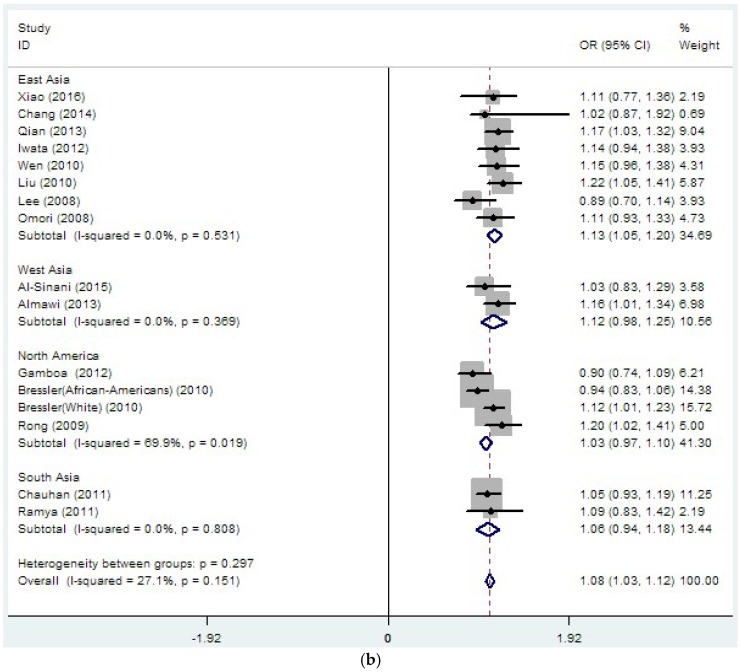
The stratified analysis results of rs8050136 grouped by region: (**a**) without; and (**b**) with adjustment for BMI.

**Figure 4:**
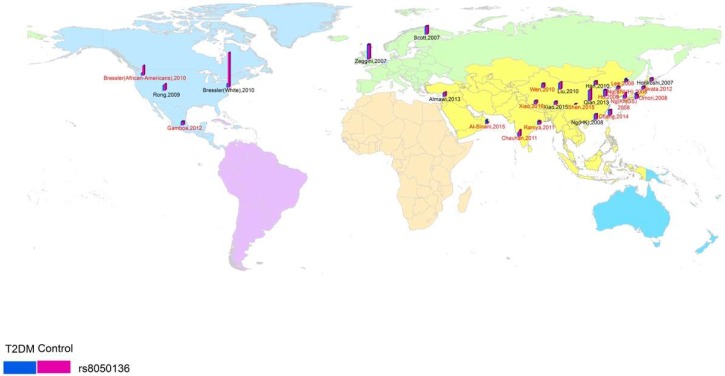
Geographic distribution of selected studies exploring the association between rs8050136 and T2DM risk. Blue bars indicate T2DM patients while pink bars indicate controls; the height of bars is proportional to sample size. Studies in black text represent those that showed a significant association between the SNP and T2DM risk. Studies in red text indicate no significant association.

For rs8050136, a total of 33,889 T2DM cases and 45,490 controls were included in the final data analysis. The overall results showed a significant association between rs8050136 and T2DM risk (OR = 1.14, 95% CI 1.10–1.18, *p* (*z*-test) < 0.001, *I*^2^ = 37.4%) (Table 2, Figure 2a), with the association remaining statistically significant after adjustment for BMI (OR = 1.08, 95% CI 1.03–1.12, *p* (*z*-test) < 0.001, *I*^2^ =27.1%) (Table 2, Figure 2b). To more clearly understand the association between rs8050136 and T2DM in different regions, we performed the subgroup analyses by region. Consequently, without BMI adjustment, a significant association between rs8050136 and T2DM was uncovered in East Asia (OR = 1.15, 95% CI 1.10–1.20), West Asia (OR = 1.17, 95% CI 1.05–1.29) and Europe (OR = 1.19, 95% CI 1.14–1.25) (Table 2, Figure 3a), with no such association in North America (OR = 1.06, 95% CI 0.93–1.19) or South Asia (OR = 1.19, 95% CI 0.91–1.48). After adjustment for BMI, significant association was only observed in East Asia (OR = 1.13, 95% CI 1.05–1.20) (Table 2, Figure 3b). More importantly, as seen in Figure 4, the majority of studies on rs8050136 were distributed in East Asia. Several other studies were scattered throughout Europe, Northern America, South Asia and West Asia. More data for these regions may be required to detect an association.

For rs9939609, a total of 32,771 T2DM cases and 50,161 controls were included in the meta-analysis. The overall results indicated that rs9939609 was significantly associated with an increased risk of T2DM (OR = 1.15, 95% CI 1.11–1.19, *p* (*z*-test) < 0.001, *I*^2^ = 53.2%) (Table 2, Figure S1a). After adjustment for BMI, the association remained statistically significant (OR = 1.11, 95% CI 1.05–1.17, *p* (*z*-test) < 0.001, *I*^2^ = 56.1%) (Table 2, Figure S1b). Due to the heterogeneity that existed between studies, we performed stratified analyses grouped by region. In the subgroup analyses, similar results were found in East Asia (without BMI adjustment: OR = 1.11, 95% CI 1.05–1.17; with BMI adjustment: OR = 1.11, 95% CI 1.02–1.20) and South Asia (without BMI adjustment: OR = 1.19, 95% CI 1.10–1.29; with BMI adjustment: OR = 1.19, 95% CI 1.06–1.31), whereas no such association existed between rs9939609 and T2DM in North America (without BMI adjustment: OR = 1.11, 95% CI 0.89–1.32; with BMI adjustment: OR = 1.02, 95% CI 0.81–1.22) (Table 2, Figure S2). Additionally, in Europe, a significant association between rs9939609 and T2DM was observed without BMI adjustment (OR = 1.18, 95% CI 1.14–1.22), whereas no association was uncovered with BMI adjustment (OR = 1.11, 95% CI 0.93–1.29). Similar to the distributions of rs8050136 studies, the geographic distribution of researches on rs9939609 were concentrated in East Asia and South Asia, where the association was found to be significant.

**Figure 5:**
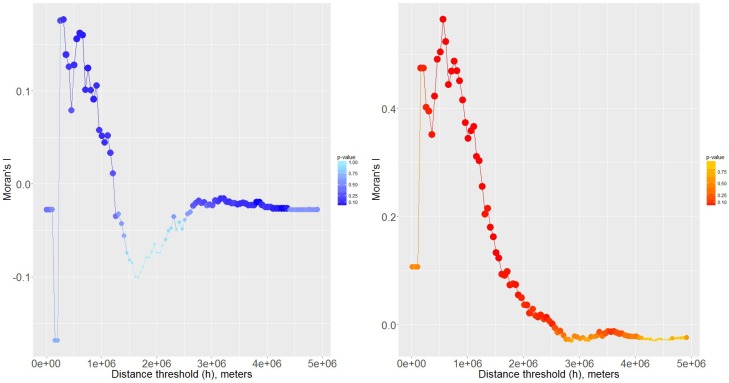
Spatial autocorrelation analysis of rs8050136, rs9939609 and T2DM by Moran’s I. Blue indicates the results of rs8050136 and red indicates the results of rs9939609; the size and shade of the circles are proportional to the significance of Moran’s I.

As illustrated in Figure 5, when the spatial scale was smaller than 1,000,000 meters, there was significant positive spatial autocorrelation in terms of both rs9969309 and rs8050136. It turned out that in relative small spatial scale (*h* < 1,000,000 meters), the studies with significant correlations tended to be clustered, which indicated that the correlation between rs9969309 and rs8050136, and T2DM risk was strongly associated with the geographic factors. With the *h* increasing, Moran’s *I* showed no positive spatial autocorrelation of these two SNPs and T2DM risk, which meant we cannot reject the null hypothesis of completed spatial randomness. Our results follow Tobler’s first law of geography: “Everything is related to everything else, but near things are more related than distant things” (pp.236, [56]). It seemed that in Asia, there was a strong positive-positive (significant-significant) spatial autocorrelation while in Europe there may be some negative-negative (non-significant-non-significant) spatial autocorrelation. In North America, the spatial autocorrelation was not significant, maintaining a relatively random spatial pattern.

For rs1421085 and rs17817499, a total of 4,285 T2DM cases with 16,279 controls and 2,634 T2DM cases with 15,482 controls, respectively, were identified for data analysis. The results indicated that neither rs1421085 nor rs17817499 were associated with T2DM, independent of BMI adjustment (Table 2, Figures S3 and S4). Compared with rs9939609 and rs8050136, studies that focused on rs1421085 and rs17817499 were relatively fewer and were distributed in North America and North Africa.

### 3.3. Sensitivity Analyses

To assess the stability of the combined results obtained by excluding studies of unknown HWE in controls [7,25], a sensitivity analysis was conducted (Figure S5). The analysis confirmed that the rs9939609 polymorphism conferred a predisposition to T2DM.

### 3.4. Assessment of Publication Bias

To evaluate the publication bias, we performed Begg’s test and Egger’s test. The results showed that there was no publication bias for the associations between the four *FTO* polymorphisms and T2DM risk (*p* > 0.05 for Begg’s test and Egger’s test) (Table S1).

## 4. Discussion

Our meta-analysis and spatial analysis are based on a large sample size, including over 60,000 and 90,000 subjects for rs9939609 and rs8050136, respectively, spanning regions across Asia, Europe and Northern America. In line with previous meta-analyses of Asian populations [14,36,45], we further demonstrated a strong association between rs9939609 and rs8050136, and T2DM regardless of adjustment for BMI (Table 2, Figure 2 and Figure 3, Figures S1 and S2). Notably, the associations are region-related.

Indeed, some statistics such as Moran’s I [16,17], and local indicators of spatial autocorrelation (LISA) [57] can be used to quantitatively study spatial autocorrelation. However, due to obstacles including the modifiable areal unit problem (MAUP) (i.e., some papers only provide a country location while some papers have the city location) and the low data volume, it is difficult to perform spatial statistics for rs1421085 and rs17817499 to further explore the spatial pattern. Nevertheless, our data still indicate the geographic factor may play an important role in the correlations between T2DM risk and rs8050136 (Figure 4 and Figure 5), rs9939609 (Figure 5).

Initially, the articles we reviewed contained more than 10 types of *FTO* SNPs in T2DM patients and controls, but we eventually chose the four most common SNPs, namely rs9939609, rs8050136, rs1421085 and rs17817499. All four SNPs are located in intron 1 of the *FTO* gene, a region of strong linkage disequilibrium [40]. Some studies have found no direct connection between the variants and *FTO* expression or function [9], while other studies have suggested that variants of *FTO* play an important role in regulating body weight and fat mass by influencing food intake [6]. A recent report revealed that SNPs in *FTO* could influence obesity by altering the expression of the adjacent genes *IRX3* and *RPGRIP1L* [58]. Although mechanisms regarding how these noncoding variants affect T2DM are not yet clear, Smemo et al. have demonstrated that variants within *FTO* can form long-range functional connections with *IRX3*, representing a determinant of body mass and composition [59]. Additionally, recent studies have suggested hepatic FTO contributes to glucose homeostasis [60,61,62], indicating that FTO may play a role in the regulation of carbohydrate metabolism.

Of note, the overall heterogeneity of rs9939609 increased slightly after BMI adjustment (*I*^2^ = 53.2%, *p* < 0.001 without BMI adjustment vs. *I*^2^ = 56.1%, *p* = 0.003 with BMI adjustment) (Table 2), suggesting that BMI may not primarily account for heterogeneity. To this end, we performed additional subgroup analyses by region and found that heterogeneity still existed in the group of North America and South Asia independent of BMI adjustment. We then excluded each study in South Asia and North America and performed subgroup analyses, respectively. When omitting studies by Fawwad et al. or Chauhan et al. in South Asia, as well as Bressler et al. (African-Americans) in North America [24,32,34], the heterogeneity disappeared in the South Asian (*I*^2^ = 34.6%, *p* = 0.141 and *I*^2^ = 37.2%, *p* = 0.121) and North American (*I*^2^ = 0.0%, *p* = 0.667) subgroups, respectively, without BMI adjustment (Table S2). Of note, the heterogeneity showed no change by removing other studies in South Asian or North American subgroup. Alternatively, only removing the study by Ali et al. [27], heterogeneity in the South Asian subgroup also attenuated sharply (*I*^2^ = 20.3%, *p* = 0.288) after adjustment for BMI (Table S2). These results demonstrated that these studies mentioned above were the main source of heterogeneity in South Asia and North America. Unlike rs9939609, owing to the low data volume of the studies, the heterogeneity in rs1421085 and rs17817499 showed no change by subgroup analyses.

BMI is widely considered as a confounder of T2DM risk. In this study, the overall associations between the four SNPs and T2DM risk were not affected by BMI adjustment. (Table 2), indicating that the overall associations were BMI-independent. Nevertheless, in Europe for rs9939609 and West Asia for rs8050136, the BMI adjustment altered the associations (Table 2). In agreement with previous reports [11,12], our data showed that rs9939609 was also associated with T2DM risk somewhat independently of BMI in East and South Asia as well as in Europe. Interestingly, different regions showed different associations between rs9939609 and rs8050136, and T2DM risk, demonstrating that the associations were region-dependent. Generally, a race/ethnicity population might live in the same region in most of the non-immigrant countries. Thus, our results might reflect the influence of different races/ethnicities to some extent.

The rs9939609 was the first SNP discovered within the *FTO* gene that showed a strong association with BMI and as such is the most widely investigated SNP of *FTO* [63]. Additionally, the A allele of rs9939609 is known to indicate a predisposition to obesity, T2DM, polycystic ovary syndrome (PCOS) and some cancers [41,64,65]. Our results of rs9939609 are not only consistent with earlier reports [11,12,13,14], but also include more recent studies with greater geographical coverage [7,8,9,22,23,34] (Table 2, Figure S2), providing stronger evidence for these associations. Similarly, rs8050136 was also found to function as a susceptible SNP to rs9939609-related diseases. Unlike rs9939609 and rs8050136, studies on rs1421085 and rs17817499 are scarce, and have limited regional coverage; lack of association maybe due to smaller sample size and less studies involved.

The study we present here still possesses several limitations. First, a large proportion of the studies focused on Asian populations, with European and Northern American populations only accounting for a small part. Second, there were relatively few studies on rs1421085 and rs17817499, which may lead to bias in negative results (Table 2, Figures S3 and S4). Lastly, except for BMI, we used genotype data without considering other possible confounders (such as age and sex) or gene-gene and gene–environment interactions. Although BMI is widely used to measure obesity, it has been suggested that different criteria (not necessarily > 30) may be used in different ethnic populations. Adiposity (or specific distribution of fat) rather than body weight (or BMI) may play a critical role in the regulation of insulin sensitivity and the development of diabetes. This may lead to an inconsistency in the effect of BMI on the association between *FTO* variants and T2DM risk. Therefore, further studies that adjust for more concomitant factors and cover more regions should be conducted.

## 5. Conclusions

The spatial analysis and meta-analysis showed that the associations between genetic polymorphisms in *FTO* and T2DM are region-related and that shedding light on spatial variations can provide new insights into well-established relationships. The rs9939609 and rs8050136 SNPs contributed to an increased risk of T2DM, which could provide new solutions for T2DM prevention and therapy. This study presented an initial step in spatial analysis for genetic and regional factors in the development of diabetes, although more work remains to be done before we can understand the impact of genetics, environment, geography, BMI and fat distribution on diabetes as well as how these associations may vary across space.

## Figures and Tables

**Table 1:** Characteristics of the included studies.

First Author	Year	Region	Sample Size		Risk Allele Frequency		HWE	Ref.
			T2DM	Control	T2DM	Control		
**rs9939609**								
Phani	2016	South Asia	518	518	0.54	0.59	NA	[7]
Xiao	2016	East Asia	879	895	0.341	0.295	yes	[22]
Xiao	2015	East Asia	849	873	0.336	0.292	yes	[8]
Shen	2015	East Asia	81	80	0.125	0.106	yes	[9]
Al-Sinani	2015	West Asia	992	294	0.48	0.435	yes	[23]
Fawwad	2015	South Asia	296	198	0.588	0.391	yes	[24]
Raza	2014	South Asia	101	97	0.406	0.376	NA	[25]
Bazzi	2014	South Asia	81	95	0.525	0.542	yes	[10]
Kalnina	2013	Europe	974	1075	0.501	0.438	yes	[26]
Ali	2013	South Asia	1583	1317	0.362	0.304	yes	[27]
Binh	2012	East Asia	98	251	0.255	0.181	yes	[28]
Iwata	2012	East Asia	722	758	0.206	0.182	yes	[29]
Rees(COBRA)	2011	South Asia	385	1281	0.336	0.294	yes	[30]
Rees(UKADS/DGP)	2011	South Asia	1568	1177	0.329	0.298	yes	[30]
Huang	2011	East Asia	591	1200	0.299	0.305	yes	[31]
Chauhan	2011	South Asia	2361	2755	0.35	0.34	yes	[32]
Cruz	2010	North America	519	547	0.252	0.212	yes	[33]
Bressler	2010	North America	655	2685	0.463	0.483	yes	[34]
(African-American)								
Bressler(white)	2010	North America	988	9915	0.465	0.443	yes	[34]
Liu	2010	East Asia	1774	1984	0.136	0.117	yes	[35]
Yajnik	2009	South Asia	1453	1361	0.353	0.3	yes	[36]
Legry	2009	Europe	283	2601	0.456	0.42	yes	[37]
Sanghera	2008	South Asia	513	353	0.363	0.31	yes	[38]
Horikawa	2008	East Asia	1849	1578	0.209	0.205	yes	[39]
Chang	2008	East Asia	735	726	0.132	0.127	yes	[40]
Omori	2008	East Asia	1621	1053	0.209	0.195	yes	[2]
Horikoshi	2007	East Asia	864	864	0.216	0.192	yes	[41]
Zeggini.	2007	Europe	5681	8284	0.435	0.394	yes	[42]
Frayling	2007	Europe	3757	5346	NA	NA	yes	[6]
**rs8050136**								
Xiao	2016	East Asia	879	895	0.313	0.275	yes	[22]
Xiao	2015	East Asia	849	873	0.308	0.274	yes	[8]
Shen	2015	East Asia	88	80	0.114	0.106	yes	[9]
Al-Sinani	2015	West Asia	992	294	0.458	0.425	yes	[23]
Chang	2014	East Asia	1502	1518	0.127	0.124	yes	[43]
Almawi	2013	West Asia	995	1195	0.487	0.551	yes	[44]
Qian	2013	East Asia	2898	3262	0.127	0.103	yes	[45]
Gamboa	2012	North America	1027	990	0.194	0.2	yes	[46]
Iwata	2012	East Asia	724	763	0.205	0.183	yes	[29]
Chauhan	2011	South Asia	1106	1800	0.35	0.34	yes	[32]
Ramya	2011	South Asia	1001	851	0.14	0.107	yes	[47]
Han	2010	East Asia	1007	995	0.13	0.11	yes	[48]
Bressler	2010	North America	657	2728	0.425	0.44	yes	[34]
(African-American)								
Bressler(White)	2010	North America	984	9873	0.444	0.402	yes	[34]
Wen	2010	East Asia	1165	1136	0.134	0.119	yes	[49]
Liu	2010	East Asia	1748	2015	0.139	0.117	yes	[35]
Hu	2009	East Asia	1849	1785	0.13	0.118	yes	[50]
Rong	2009	North America	1472	1825	0.151	0.136	yes	[51]
Lee	2008	East Asia	886	501	0.129	0.14	yes	[52]
Ng(HK)	2008	East Asia	1481	1530	0.156	0.136	yes	[53]
Ng(SNUH)	2008	East Asia	761	632	0.138	0.122	yes	[53]
Ng(KHGS)	2008	East Asia	799	1516	0.124	0.118	yes	[53]
Omori	2008	East Asia	1616	1060	0.208	0.194	yes	[2]
Horikoshi	2007	East Asia	857	861	0.238	0.2	yes	[41]
Zeggini	2007	Europe	4207	4111	0.44	0.39	yes	[42]
Scott	2007	Europe	2339	2401	0.406	0.381	yes	[54]
**rs1421085**								
Cauchi(Morocco)	2012	North Africa	1193	1095	0.395	0.356	yes	[55]
Cauchi(Tunisia)	2012	North Africa	1446	942	0.41	0.407	yes	[55]
Bressler	2010	North America	657	2725	0.084	0.112	yes	[34]
(African-American)								
Bressler(White)	2010	North America	989	9893	0.451	0.41	yes	[34]
**rs17817499**								
Almawi	2013	West Asia	995	1195	0.517	0.557	yes	[44]
Bressler	2010	North America	653	2700	0.376	0.396	yes	[34]
(African-American)								
Bressler(White)	2010	North America	986	9948	0.443	0.403	yes	[34]

T2DM, Type 2 diabetes mellitus; HWE, Hardy–Weinberg equilibrium; COBRA, Control of Blood Pressure and Risk Attenuation; UKADS/DGP, UK Asian Diabetes Study/Diabetes Genetics in Pakistan; HK, Hong Kong; SNUH, Seoul National University Hospital; KHGS, Korean Health and Genome Study.

**Table 2:** Meta-analysis of fat mass and obesity-associated (*FTO*) single-nucleotide polymorphisms (SNPs) and T2DM risk.

SNP	No. of Study (T2DM/Control)	Without BMI Adjustment				With BMI Adjustment			
		OR	*p_z_* ^a^	*I*^2^ (%)	*P_H_* ^b^	OR	*p_z_* ^a^	*I*^2^ (%)	*P_H_* ^b^
		(95% CI)				(95% CI)			
**All**									
rs9939609	29	1.15	0	53.2	0	1.11	0	56.1	0.003
	(32771/50161)	(1.11–1.19)				(1.05–1.17)			
rs8050136	26	1.14	0	37.4	0.032	1.08	0	27.1	0.151
	(33889/45490)	(1.10–1.18)				(1.03–1.12)			
rs1421085	4	1.05	0.48	80.6	0.001	1.02	0.755	78.2	0.003
	(4285/16279)	(0.91–1.21))				(0.88–1.19)			
rs17817499	3	1.09	0.271	82.7	0.003	1.05	0.539	80	0.007
	(2634/15482)	(0.93–1.28)				(0.90–1.23)			
**East Asia**									
rs9939609	11	1.11	0	19.5	0.257	1.11	0	0	0.535
	(10063/10262)	(1.05–1.17)				(1.02–1.20)			
rs8050136	15	1.15	0	0	0.789	1.13	0	0	0.531
	(19109/19422)	(1.10–1.20)				(1.05–1.20)			
**North America**									
rs9939609	3	1.11	0	85.4	0.001	1.02	0	85.7	0.008
	(2162/14790)	(0.89–1.32)				(0.81–1.22)			
rs8050136	4	1.06	0	74.1	0.009	1.03	0	69.9	0.019
	(4140/17082)	(0.93–1.19)				(0.97–1.10)			
**Europe**									
rs9939609	4	1.18	0	0	0.49	1.11	0	75.6	0.043
	(10695/17306)	(1.14–1.22)				(0.93–1.29)			
rs8050136	2	1.19	0	46.5	0.172	NA	NA	NA	NA
	(8020/10685)	(1.14–1.25)							
**South Asia**									
rs9939609	10	1.19	0	58.6	0.01	1.19	0	69.7	0.01
	(8859/9152)	(1.10–1.29)				(1.06–1.31)			
rs8050136	2	1.19	0	68	0.077	1.06	0	0	0.808
	(2107/2651)	(0.91–1.48)				(0.94–1.18)			
**West Asia**									
rs8050136	2	1.17	0	0	0.76	1.12	0	0	0.369
	(1987/1489)	(1.05–1.29)				(0.98–1.25)			

^a^ *p* value for *z*-test; ^b^ *p* value for χ^2^-test based Q test; BMI, body mass index; OR, odds ratio; CI, confidence interval; NA, not available.

## Supplementary Material

The following can be found online at www.mdpi.com/2073-4425/8/2/70/s1, Table S1. Publication bias of FTO SNPs; Table S2. Heterogeneity for rs9939609 in South Asia and North America subgroups after excluding each study; Figure S1. Meta-analysis for the associations between rs9939609 and T2DM risk (a) without and (b) with adjustment for body mass index (BMI); Figure S2. The stratified analysis results of rs9939609 grouped by region (a) without and (b) with adjustment for body mass index (BMI); Figure S3. Meta-analysis for the associations between rs1421085 and T2DM risk (a) without and (b) with adjustment for body mass index (BMI); Figure S4. Meta-analysis for the associations between rs17817499 and T2DM risk (a) without and (b) with adjustment for body mass index (BMI); Figure S5. Sensitivity analysis of rs9939609 by excluding studies with an unknown Hardy-Weinberg equilibrium (HWE) in controls.
