# Supplementary material for: FTO Genotype and Type 2 Diabetes Mellitus: Spatial Analysis and Meta-Analysis of 62 Case-Control Studies from Different Regions
Source: Genes (Basel). 2017 Feb 11;8(2):70. doi: 10.3390/genes8020070 (PMC5333059; doi:10.3390/genes8020070)
Supplement: Supplementary file 1 [file genes-08-00070-s001.pdf]

FTO genotype and type 2 diabetes mellitus: spatial analysis and meta-analysis of 62 case-control studies from different regions

Ying Yang [1,†], Boyang Liu [2,†], Wei Xia [3,†], Jing Yan [4], Huan-Yu Liu [5], Ling Hu [6], Song-Mei Liu [1,*]

[1] Center for Gene Diagnosis, Zhongnan Hospital of Wuhan University, Donghu Road 169#, Wuhan 430071, China; yangying0109@whu.edu.cn
[2] Department of Geography, Wilkeson Hall, State University of New York at Buffalo, Buffalo, NY 14261, USA; bliu24@buffalo.edu
[3] Department of Clinical Laboratory, Wuhan Children's Hospital (Wuhan Maternal and Child Healthcare Hospital), Tongji Medical College, Huazhong University of Science & Technology, Wuhan 430016, China; 18971319110@163.com
[4] Hubei Meteorological Information and Technology Support Center, Wuhan 430074, China; yanjing619@hotmail.com
[5] Department of Clinical Medicine, Hubei University of Medicine, Hubei 442000, China; gutentag95@sina.com
[6] Department of Neurology, Wuhan Children's Hospital (Wuhan Maternal and Child Healthcare Hospital), Tongji Medical College, Huazhong University of Science & Technology, Wuhan 430016, China; m18372622675@163.com
* Correspondence: smliu@whu.edu.cn; Tel./Fax: +86-27-6781-2610
† These authors contributed equally to this work.

Table S1. Publication bias of *FTO* SNPs.

SNP	Without BMI adjustment		With BMI adjustment	
	p_B [a]	p_E [b]	p_B [a]	p_E [b]
rs9939609	0.940	0.484	0.418	0.109
rs8050136	0.159	0.469	0.280	0.515
rs1421085	0.174	0.114	0.174	0.152
rs17817499	0.117	0.612	0.602	0.920

[a] *p* value for Begg's test; [b] *p* value for Egger's test.

Table S2. Heterogeneity for rs9939609 in South Asia and North America subgroups after excluding each study.

Excluded study	Subgroup	Heterogeneity	
		I^2(%)	*P*
**Without BMI adjustment**			
Phani	South Asia	63.0	0.006
Fawwad	South Asia	34.6	0.141
Raza	South Asia	63.2	0.005
Bazzi	South Asia	60.9	0.009
Ali	South Asia	57.7	0.015
Rees (COBRA)	South Asia	62.9	0.006
Rees (UKADS/DGP)	South Asia	63.2	0.005

Chauhan	South Asia	37.2	0.121
Yajnik	South Asia	58.9	0.013
Sanghera	South Asia	62.3	0.007
Cruz	North America	91.4	0.001
Bressler(African-Americans)	North America	0.0	0.667
Bressler(White)	North America	82.2	0.018
**With BMI adjustment**			
Ali	South Asia	20.3	0.288
Rees (COBRA)	South Asia	77.1	0.004
Rees (UKADS/DGP)	South Asia	77.3	0.004
Chauhan	South Asia	54.0	0.089
Yajnik	South Asia	75.3	0.007

Studies in red text represent the heterogeneity disappeared after removing this study.

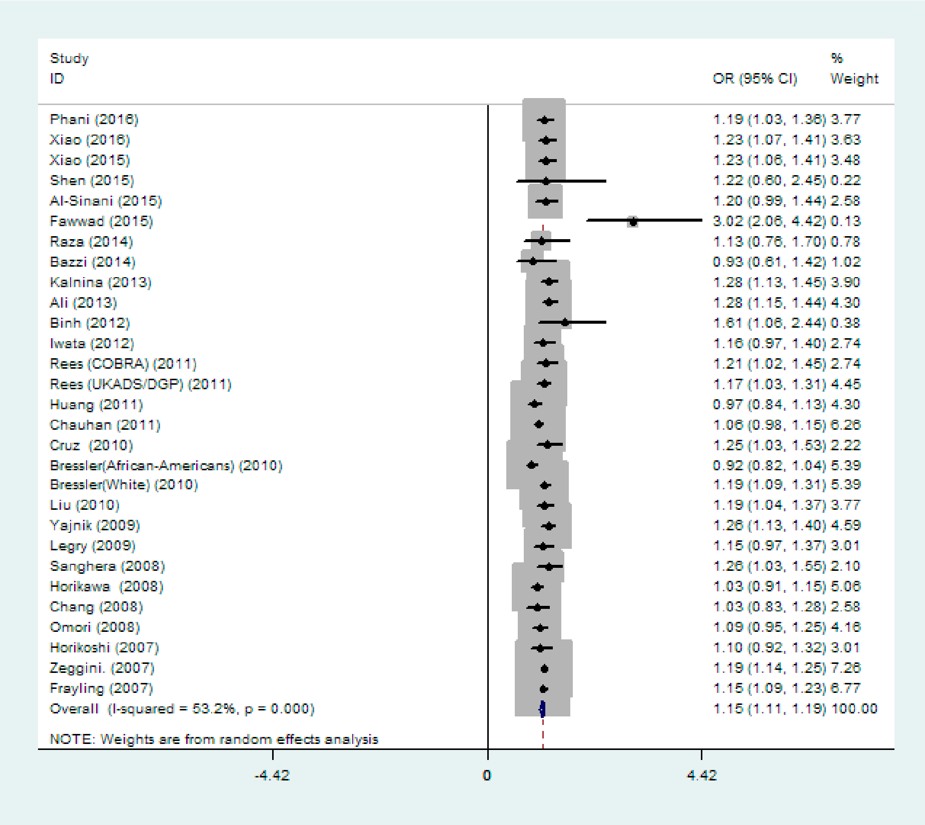

(a)

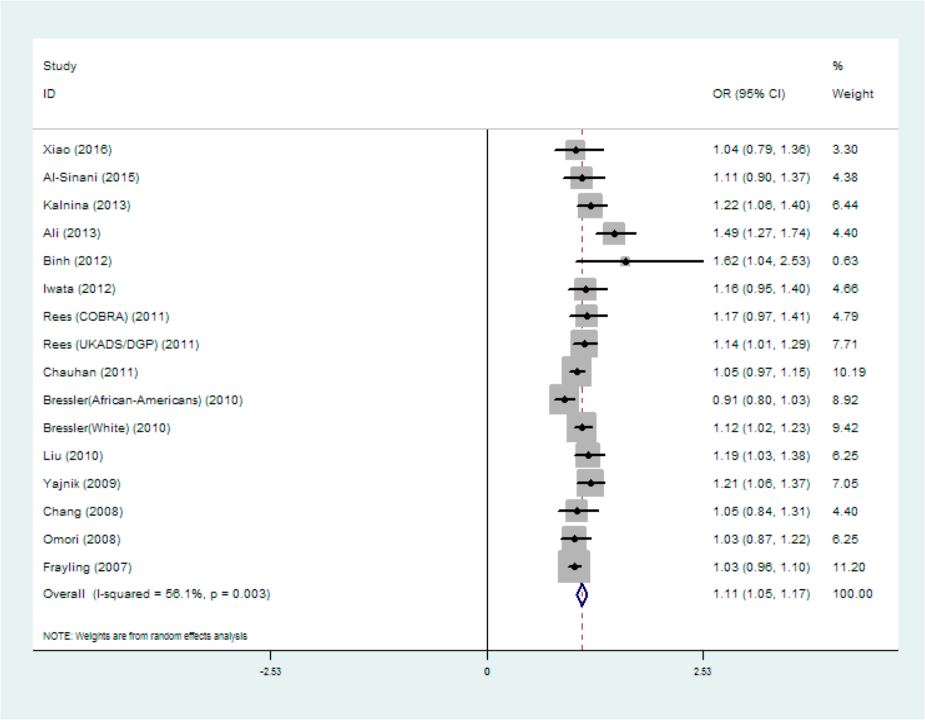

(b)

Figure S1. Meta-analysis for the associations between rs9939609 and T2DM risk **(a)** without and **(b)** with adjustment for body mass index (BMI).

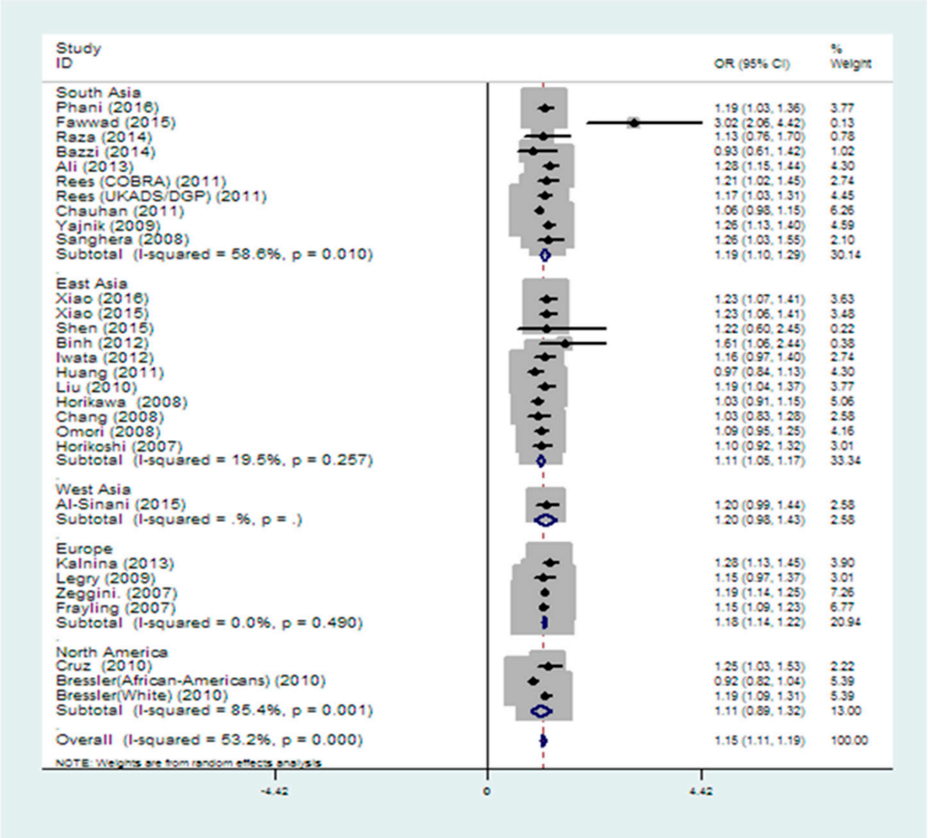

(a)

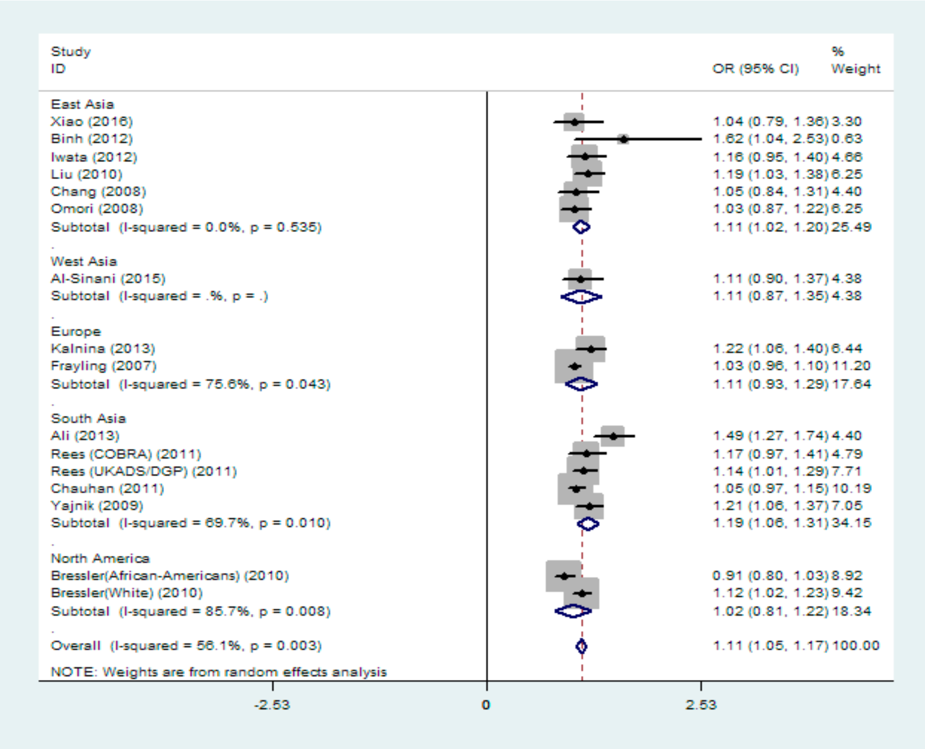

(b)

Figure S2. The stratified analysis results of rs9939609 grouped by region **(a)** without and **(b)** with adjustment for body mass index (BMI).

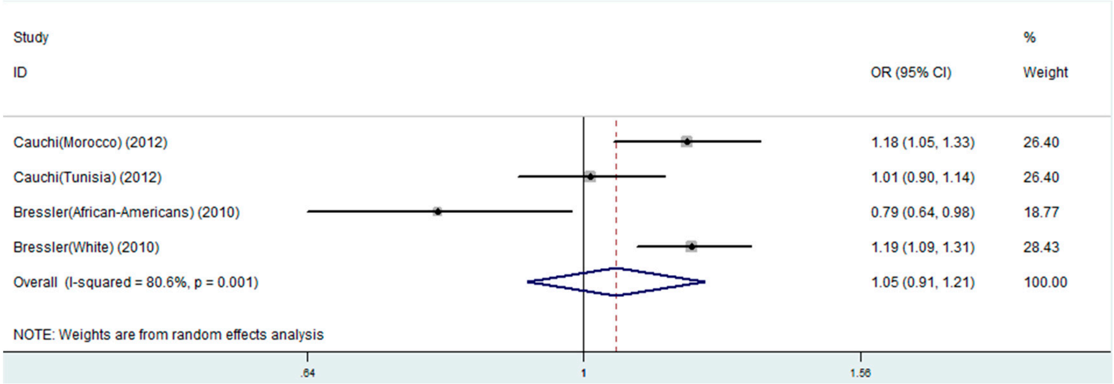

(a)

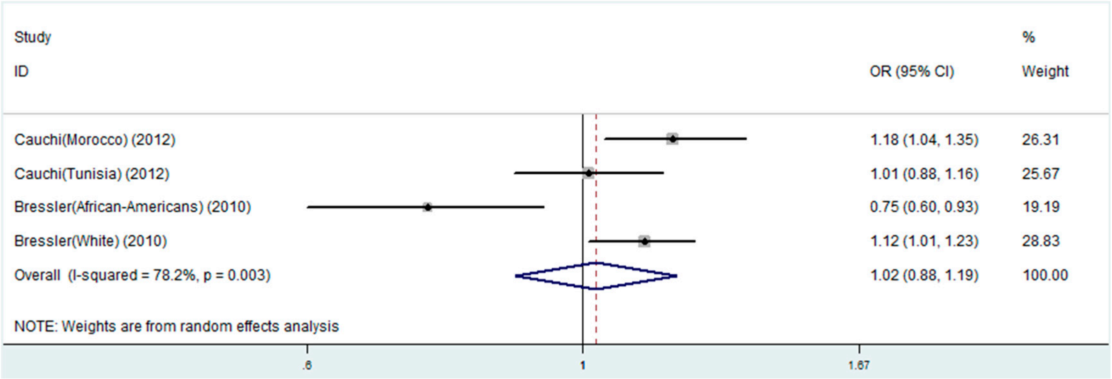

(b)

Figure S3. Meta-analysis for the associations between rs1421085 and T2DM risk **(a)** without and **(b)** with adjustment for body mass index (BMI).

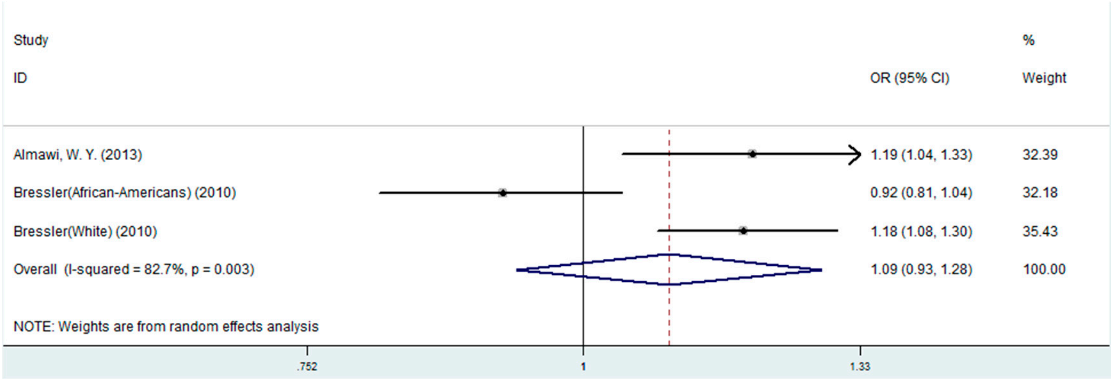

(a)

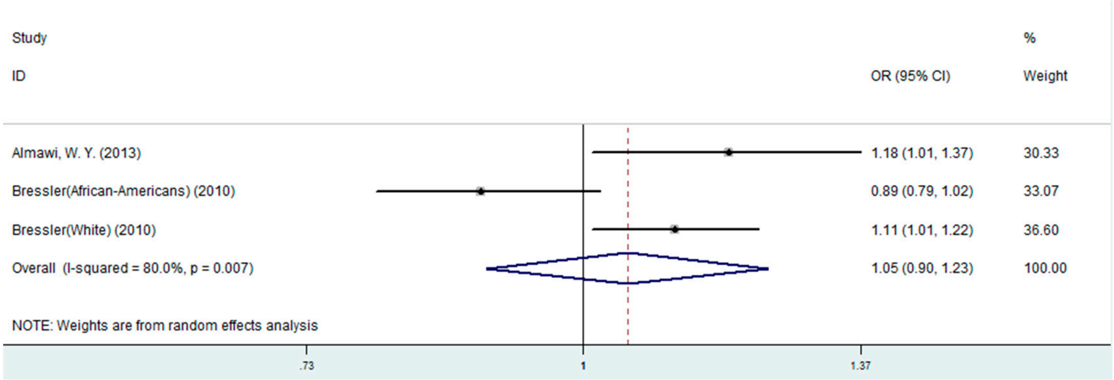

(b)

Figure S4. Meta-analysis for the associations between rs17817499 and T2DM risk **(a)** without and **(b)** with adjustment for body mass index (BMI).

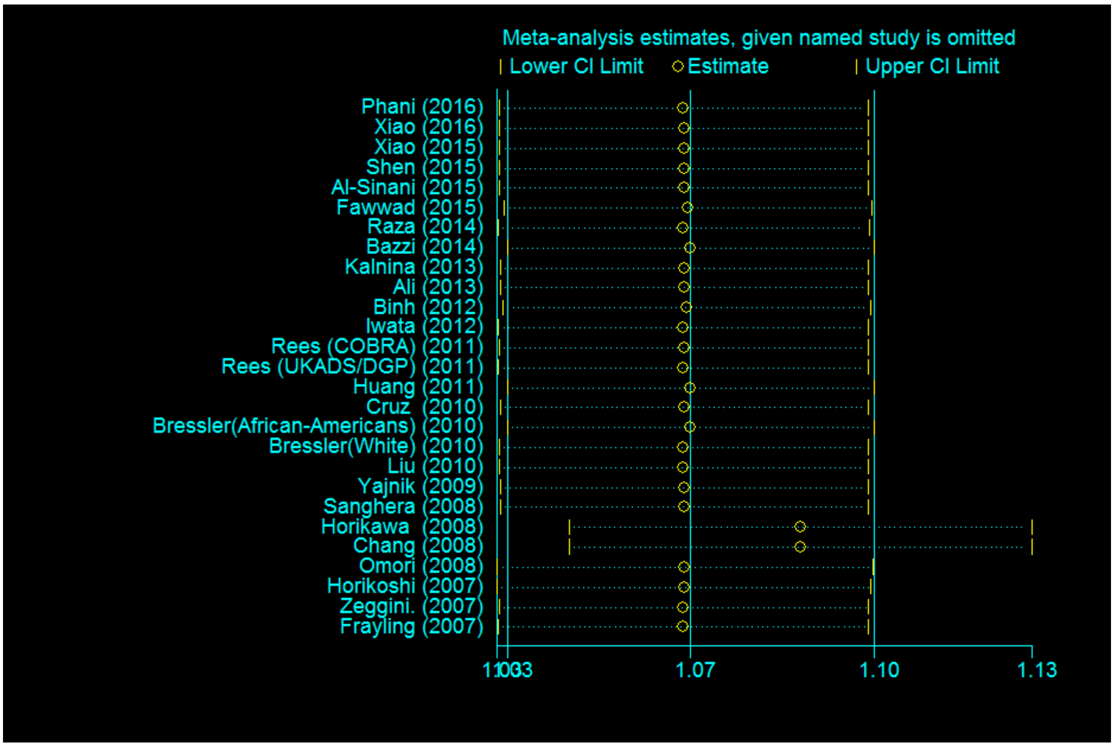

Figure S5. Sensitivity analysis of rs9939609 by excluding studies with an unknown Hardy-Weinberg equilibrium (HWE) in controls [1,2].

References

1. Phani, N.M.; Vohra, M.; Rajesh, S.; Adhikari, P.; Nagri, S.K.; D'Souza, S.C.; Satyamoorthy, K.; Rai, P.S. Implications of critical *PPARγ2*, *ADIPOQ* and *FTO* gene polymorphisms in type 2 diabetes and obesity-mediated susceptibility to type 2 diabetes in an Indian population. *Mol. Genet. Genomics* **2016**, *291*, 193–204.
2. Raza, S.T.; Abbas, S.; Ahmad, A.; Ahmed, F.; Zaidi, Z.H.; Mahdi, F. Association of glutathione-s-transferase (GSTM1 and GSTT1) and FTO gene polymorphisms with type 2 diabetes mellitus cases in Northern India. *Balk. J. Med. Genet.* **2014**, *17*, 47–54.

© 2017 by the authors; licensee MDPI, Basel, Switzerland. This article is an open access article distributed under the terms and conditions of the Creative Commons Attribution (CC BY) license (http://creativecommons.org/licenses/by/4.0/).
